# Availability of secondary prevention services after stroke in Europe: An ESO/SAFE survey of national scientific societies and stroke experts

**DOI:** 10.1177/2396987318816136

**Published:** 2018-11-27

**Authors:** A Webb, MR Heldner, D Aguiar de Sousa, EC Sandset, G Randall, Y Bejot, B van der Worp, V Caso, U Fischer, On behalf of the ESO-SAFE Secondary Prevention Survey Steering Group

**Affiliations:** 1 Centre for Prevention of Stroke and Dementia, University of Oxford, Oxford, UK; 2 Department of Neurology, Inselspital, Bern University Hospital, University of Bern, Bern, Switzerland; 3 Department of Neurosciences and Mental Health (Neurology), Hospital de Santa Maria, University of Lisbon, Lisbon, Portugal; 4 Department of Neurology, Oslo University Hospital, Oslo, Norway; 5 Department of Research and Development, the Norwegian Air Ambulance Foundation, Oslo, Norway; 6 Stroke Alliance for Europe, Brussels, Belgium; 7 Dijon Stroke Registry, EA7460, Pathophysiology and Epidemiology of Cerebro-Cardiovascular diseases (PEC2), University Hospital of Dijon, University of Burgundy, Dijon, France; 8 Department of Neurology and Neurosurgery, Brain Center Rudolf Magnus, University Medical Center Utrecht, Utrecht, the Netherlands; 9 Santa Maria della Misericordia Hospital, University of Perugia, Perugia, Italy

**Keywords:** Survey, Europe, stroke, secondary prevention

## Abstract

**Background:**

Recurrent stroke is associated with increased disability and cognitive impairment, but the availability of secondary prevention measures after transient ischaemic attack (TIA) or stroke in Europe is uncertain. This limits prioritisation of investment and development of national stroke strategies.

**Methods:**

National stroke representatives throughout Europe were surveyed. Consensus panels reported national data if available, or else expert opinion, estimating the availability of each intervention by quintiles of patients, dichotomised for analysis at 60%. Countries were classified into tertiles of gross domestic product per capita.

**Results:**

Of 50 countries, 46 responded; 14/45 (31%) had national stroke registries and 25/46 (54.3%) had national stroke strategies incorporating secondary prevention. Respondents reported that the majority of TIA patients were assessed by specialist services within 48 hours in 74.4% of countries, but in nine countries more than 20% of patients were seen after more than seven days and usually assessed by non-specialists (7/46 countries). Eighty percent of countries deferred blood pressure assessment to primary care, whilst lifestyle management programmes were commonly available in only 46% of countries. Although basic interventions were widely available, interventions frequently not available to more than 60% of patients included: ambulatory cardiac monitoring (40% countries); prescription (26%) and continuation (46%) of statins; blood pressure control at follow-up (44%); carotid endarterectomy within one month (15%); face-to-face follow-up in hospital (33%); direct oral anticoagulants (21%). Gross domestic product per capita and reimbursement of interventions were the commonest predictors of availability of interventions.

**Conclusions:**

Provision of secondary prevention varied, with gaps in care prevalent throughout Europe, particularly in lower income countries.

## Introduction

Stroke causes more than one million deaths per year and is the second commonest cause of death and the leading cause of long-term disability in Europe.^1,2^ The annual cost of stroke in Europe is estimated to be €20 billion for direct care, €16 billion for informal care and €9 billion due to loss of productivity.^3^ Recurrent major cardiovascular events occur in more than 12% of patients over five years, even in patients receiving excellent evidence-based treatment in affluent countries^4^ and are associated with poor rehabilitation outcomes,^5^ physical disability and cognitive dysfunction.^6^ Optimal provision of secondary prevention has the potential to reduce recurrent events by up to 80%,^3^ but this requires rapid assessment, appropriate treatment and ongoing follow-up to ensure efficacy and adherence to treatments.^7^

Despite the morbidity, mortality and economic costs of recurrent stroke, and the cost-effectiveness of secondary prevention,^8^ information regarding the provision of secondary prevention services is lacking. Published registries focus upon acute stroke management and hospital-based care, with limited monitoring of ongoing secondary prevention, paralleling the clinical and political focus on acute stroke.^9^ This lack of published data is particularly severe in less affluent countries in Europe, preventing national comparisons across Europe and limiting our ability to identify targets to improve secondary prevention at the national and international level. The European Stroke Action Plan 2018–2030 will provide a roadmap for the development of healthcare policy, research and stroke services throughout Europe over the next decade, but this requires understanding of the current state of services and their heterogeneity across Europe. National stroke societies are ideally placed to estimate the current provision of secondary care within each European nation, either from national healthcare or research data or by providing a panel of experts to estimate provision specific to their healthcare system.

Following the methodology of the recent ESO/SAFE/ESMINT/EAN survey on provision of Acute Stroke care,^9^ the European Stroke Organisation (ESO) and the Stroke Alliance for Europe (SAFE) surveyed the leaders of national stroke societies across Europe to estimate the level of provision of secondary prevention services.

## Objectives

We aimed to estimate the availability of evidence-based secondary prevention interventions from acute assessment of TIA and minor stroke, initial treatment, through to ongoing care to identify key gaps in care provision across Europe, and to estimate the heterogeneity between nations and its determinants.

## Methods

### Study design and participants

This Europe-wide study of 50 countries (according to the World Health Organization (WHO) definition of Europe) sought consensus responses from panels of three experts in each country, coordinated by national stroke society chairs, or an ESO-nominated expert where there was no national society. Coordinators and experts (Supplementary Appendix 1) were responsible for identifying the most reliable, recent national data sources (i.e. stroke registries, healthcare data…) to answer the survey questions. In the absence of national or local stroke registries, the coordinator and experts were asked to perform best estimates by consensus, and took responsibility for the validity of the responses provided.

### Data collection

The survey was drafted by an ESO/SAFE steering committee and independently reviewed by two stroke experts (Peter Rothwell, Bo Norrving) and disseminated between 15 December 2017 and 2 February 2018 (Supplementary Appendix 2). Collected data were independently reviewed by two authors (AJSW, MRH). Where there was ambiguity and/or missing/conflicting responses, the steering committee requested clarification. Where ambiguity persisted, a single value was taken: the mid-point of a range, the mode of multiple respondents or the most conservative response. Related responses were grouped into representative variables (e.g. ‘commonest site of TIA assessment’ was derived from percentage of patients attending each of ‘acute admission,’ ‘stroke unit’, ‘TIA clinic’, ‘general medical clinic’, ‘primary care’; ‘reimbursement for advanced measures’ required reimbursement for four of: prolonged cardiac monitoring, PFO closure, DOACs, novel antiplatelets, carotid stenting, left atrial appendage closure and implantable loop recorders, with three of carotid stenting, PFO closure, DOACs and prolonged monitoring).

### Data analysis

The national incidence of ischaemic stroke was estimated from the Global Burden of Disease Report (2016).^10^ Nations were categorised into tertiles of gross domestic product (GDP) per capita.^11^ Numbers of interventional centres and procedures performed were standardised to national population estimates in 2016. Frequencies/rates were compared by chi-squared tests for categorical variables and by general linear models for associations between continuous variables. Potential determinants of availability of interventions included GDP per capita (IMF 2016, International Monetary Fund), healthcare expenditure, availability of national stroke plans, reimbursement, number of centres. Relationships were determined by chi-squared tests or logistic and ordinal regression for categorical outcomes, and general linear models for continuous outcomes. Data were analysed in Microsoft Excel 2010 and IBM SPSS 22.0. Chloropleth maps were drawn using https://mapchart.net/.

## Results

Of 50 countries, 46 responded of which 34 had the survey completed by a panel of three experts, 6 by two experts and 6 by the coordinator alone. Armenia, Belarus, Malta, and Tajikistan did not respond despite multiple requests. However, 32/45 (71.1% with one non-respondent to this question) countries reported access to some registry data, but registry characteristics varied significantly, particularly in lower income countries (Supplementary Appendix 3 Table 1). Stroke guidelines were in use in the majority of countries, most commonly ESO (38/46, 82.6%) or national stroke guidelines (38/46, 82.6%). However, 9/46 (19.6%) countries did not report specific healthcare strategies to improve secondary stroke prevention, either at the national or regional level, with only 25/46 (54.3%) reporting national strategies including secondary stroke prevention, more often in the top tertile of countries by income (Supplementary Appendix 3 Table 1).

**Table 1.** Availability of services to >60% of patients for after TIA or stroke, according to tertile of national income.

		GDP per capita			All respondents n (%)	
	N	Lower tertile n (%)	Mid-tertile n (%)	Upper tertile n (%)		p value
TIA assessment location
Hospital	45	3 (19)	6 (40)	8 (57)	17 (38)	0.09
Stroke Team	43	3 (20)	3 (21)	7 (50)	13 (30)	0.15
TIA clinic	37	0 (0)	0 (0)	1 (7)	1 (3)	0.43
General Clinic	41	2 (13)	0 (0)	0 (0)	2 (5)	0.16
Primary Care	41	0 (0)	1 (8)	0 (0)	1 (2)	0.33
TIA assessment delay
Same Day	44	4 (25)	6 (40)	4 (31)	14 (32)	0.67
Within 48 hours	44	4 (25)	1 (7)	2 (15)	7 (16)	0.38
Within 1 week	44	2 (13)	0 (0)	3 (23)	5 (11)	0.16
>1 week	44	0 (0)	1 (7)	1 (8)	2 (5)	0.55
Carotid Imaging
Ultrasound	46	7 (44)	12 (80)	8 (53)	27 (59)	0.11
CT-angiogram	46	2 (13)	4 (27)	4 (27)	10 (22)	0.54
MR-angiogram	46	3 (19)	2 (13)	2 (13)	7 (15)	0.89
2 modalities	43	1 (7)	2 (14)	3 (20)	6 (14)	0.61
Cardiac Monitoring
ECG only	42	8 (50)	4 (31)	1 (8)	13 (31)	0.05
24–48 hours	45	6 (38)	9 (60)	7 (50)	22 (49)	0.45
>48 hours	42	0 (0)	1 (7)	2 (14)	3 (7)	0.34
BP monitoring
Primary care	41	4 (29)	8 (53)	7 (58)	19 (46)	0.25
Hospital	42	3 (23)	2 (13)	2 (14)	7 (17)	0.76
Out-of-office	39	6 (50)	5 (36)	2 (15)	13 (33)	0.18
Investigated with
TTE	45	7 (47)	8 (53)	6 (40)	21 (47)	0.77
TEE	44	0 (0)	1 (7)	1 (7)	2 (5)	0.58
TCD	44	2 (13)	4 (29)	1 (7)	7 (16)	0.26
MRA/CTA	46	2 (13)	4 (27)	7 (47)	13 (28)	0.11

Groups are compared by chi-squared tests.

N: number of responses; GDP: gross domestic product; BP: blood pressure; TTE: transthoracic echocardiography; TEE: transoesophageal echocardiography; TCD: transcranial ultrasound.

Nearly all countries reported that standard medical measures and interventions for secondary prevention are reimbursed but fewer countries had reimbursement for more advanced measures (left atrial appendage closure, cardiac monitoring >72 hours, implantable loop recorders, PFO closure, direct oral anticoagulants). Reimbursement for lifestyle management programmes was not common, even in countries in the top tertile of GDP (Supplementary Appendix 3 Table 1) whilst in five lower income countries basic interventions (antiplatelets, statins, antihypertensives, vascular imaging, ECG) were not reimbursed. Overall, reimbursement was more likely in the top tertile of countries by GDP compared to the bottom two tertiles (lifestyle management 47% vs. 14%, p = 0.023; DOACs 100% vs. 63%, p = 0.008).

### Assessment

**Figure 1.**
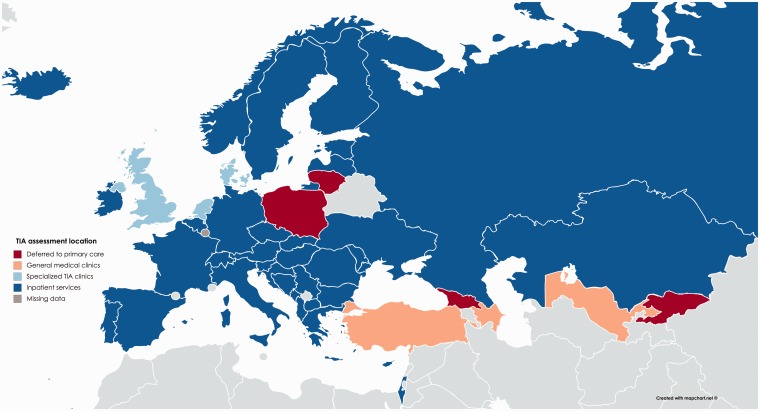
Most frequent location of assessment of patients presenting with acute TIA. Countries are coloured by the location where respondents reported that the majority of patients with acute TIA were assessed.

More than 60% of patients with a TIA were assessed by stroke specialists in higher income countries (Table 1), but four countries in the lowest tertile of GDP assessed >60% of patients in general medical clinics, whilst three countries in the lower two tertiles still deferred assessment of >20% patients to primary care (Figure 1). In 27.8% of countries, >40% of patients with a TIA were assessed by a non-specialist (Supplementary Appendix 3 Table 2). In higher income countries, assessment location varied between inpatient admission or specialist TIA clinics (Figure 1).

**Table 2.** Reported availability of treatments to >60% of patients after TIA or stroke, according to tertile of national income.

		GDP per capita			All respondentsn (%)	
	N	Lower tertile n (%)	Mid-tertile n (%)	Upper tertile n (%)		p value
Initial treatment includes
BP-lowering	46	16 (100)	14 (93)	10 (67)	40 (87)	0.015
Statin	46	9 (56)	11 (73)	14 (93)	34 (74)	0.06
Antiplatelet	46	15 (94)	15 (100)	14 (93)	44 (96)	0.60
Carotid intervention
<48 hours	36	0 (0)	1 (7)	1 (9)	2 (6)	0.61
<1 week	37	0 (0)	1 (7)	2 (17)	3 (8)	0.34
<2 weeks	39	0 (0)	1 (8)	5 (33)	6 (15)	0.043
<1 month	34	1 (9)	2 (15)	2 (20)	5 (15)	0.78
>1 month	33	5 (42)	0 (0)	0 (0)	5 (15)	0.006
Treatment at one year
BP measured	42	11 (69)	14 (100)	8 (67)	33 (79)	0.06
BP controlled	43	8 (50)	9 (64)	7 (54)	24 (56)	0.72
Lipids tested	42	6 (38)	12 (86)	6 (50)	24 (57)	0.024
Statin	43	7 (44)	8 (57)	8 (62)	23 (53)	0.60
Antiplatelet	43	14 (88)	14 (100)	11 (85)	39 (91)	0.33
Anticoagulant	42	7 (44)	10 (71)	7 (58)	24 (57)	0.31
DOAC	40	2 (13)	2 (14)	4 (36)	8 (20)	0.28
Follow-up method
Hospital	43	5 (36)	5 (36)	4 (27)	14 (33)	0.83
Specialist clinic	39	1 (8)	2 (17)	1 (7)	4 (10)	0.68
Primary care	43	5 (36)	7 (50)	10 (67)	22 (51)	0.25
No follow-up	37	2 (17)	0 (0)	0 (0)	2 (5)	0.11

Groups are compared by chi-squared tests.

N: number of responses to the question; GDP: gross domestic product; BP: blood pressure; TTE: transthoracic echocardiography; TEE: transoesophageal echocardiography; TCD: transcranial ultrasound.

**Figure 2.**
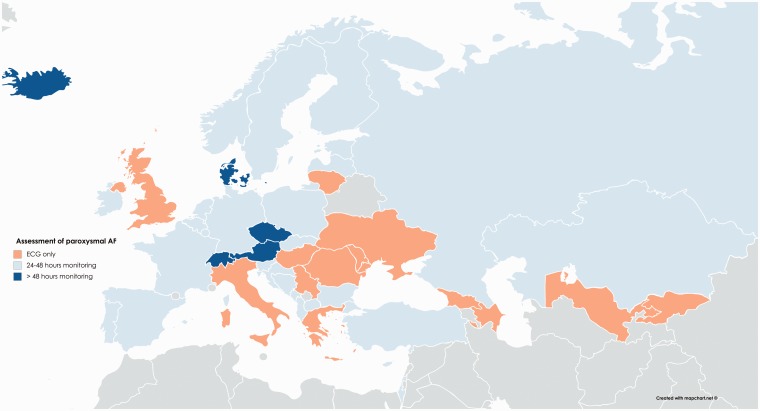
Reported form of monitoring used in >60% of patients to exclude atrial fibrillation in each nation.

Clinical guidelines in most countries recommend assessment within 48 hours for high risk TIAs (17/19, 89%) or all events (9/11, 82%), and 10% of countries recommend same day assessment, although 15/18 countries (83.3%) recommended assessment of low-risk TIAs within 7 days and 3/18 countries (16.7%) within 14 days. However, approximately 60% of countries reported seeing <20% of patients the same day whilst only 20% of countries see more than 60% in the same day (Supplementary Appendix 3 Figure 2). However, nine countries saw >20% of patients in over one week, whilst two countries took more than one week to see most patients.

Most patients in Europe undergo carotid imaging by ultrasound, with CTA or MRA being more common in a few countries (Table 1), regardless of national income. In contrast, cardiac monitoring increased with higher income (Table 1, Supplementary Appendix 3 Table 2), with extended monitoring significantly more common in the top tertile compared to the lower two tertiles (p = 0.043). However, ECG alone is the commonest method of assessing for AF in 40% of countries (Figure 2). More specialist investigations were standard (>60% patients) in some countries but were rarely performed in others (Table 1, Supplementary Appendix 3 Table 2). In particular, transoesophageal echocardiography was reported to be performed in >40% patients in six countries, whilst TCD is readily available in seven countries (Table 1) but is performed in <20% of patients in 25/44 (57%) of countries.

Blood pressure (BP) monitoring is standardly deferred to primary care (Table 1) across all countries, with only a third of countries usually performing out-of-office monitoring. The commonest target BP was 140/90 (23/41 countries, 56.1%), whilst 14/41 countries (34.1%) aimed for a BP below 130/80.

### Management

Combined lifestyle management programmes are commonly available in only half of countries (22/44), although smoking cessation and weight loss programmes are more common in the top tertile of countries by wealth (Supplementary Appendix 3 Table 1). In contrast, the majority of patients across Europe receive antiplatelets and antihypertensive medications at presentation, but statins are prescribed to <60% of patients in 26.1% of countries, particularly in lower income countries (Table 2). These differences between the use of different medication classes persist at one year but with less patients taking statins, and there is a decline in use of all agents. Despite BP and cholesterol being recorded in the majority of patients at follow-up, continuation of anticoagulants and control of BP are achieved in >60% of patients in less than 60% of countries (Table 2). However, reported use of DOACs increases in the top tertile of GDP per capita (Table 2, Supplementary Appendix 3 Table 3).

**Figure 3.**
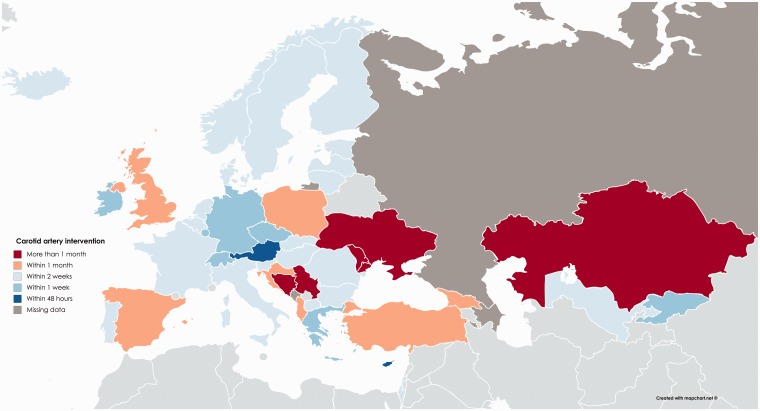
Reported delay until carotid intervention in >60% of patients.

**Figure 4.**
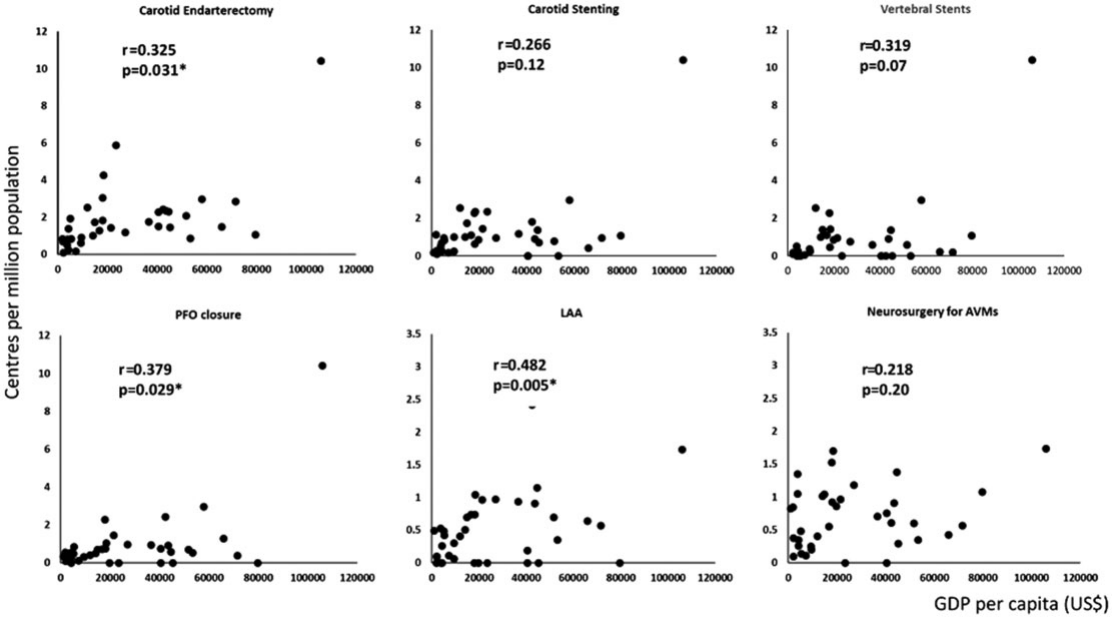
Relationship between national wealth (GDP per capita) and the number of centres offering a specific procedure. r and p values are derived from a univariate general linear regression.

Significant delays until carotid intervention remain common across Europe (Figure 3), with few countries operating within 48 hours (Austria, Cyprus) whilst five lower income countries reported that >60% of patients are not operated within one month (Figure 3). Some specialist interventions were available in most countries (Table 2), but the number of centres offering a specific procedure increased with GDP per capita (Figure 4).

### Determinants of availability of services

GDP per capita was the sole determinant of the proportion of TIA patients assessed by specialists (OR per $1000 1.05, 1.01–1.09, p = 0.02), with no significant association with health expenditure per capita, reimbursement for outpatient TIA clinics or availability of national stroke strategies. Delay until TIA assessment was not directly correlated with national income (p = 0.13), but was associated with assessment by a stroke specialist (ordinal regression OR = 0.58, 0.37–0.92, p = 0.02).

The availability of combined lifestyle modification programmes was associated with reimbursement (OR = 16.7, 1.89–146, p = 0.011) but was not associated with GDP per capita (p = 0.62). In contrast, availability of smoking cessation programmes was non-significantly correlated with reimbursement (OR = 7.8, 0.79–68.1, p = 0.06) but was associated with GDP per capita, independently of reimbursement (OR per $1000 = 1.10, 1.03–1.18, p = 0.01). Predominant use of ECG only for exclusion of atrial fibrillation was inversely associated with national income (OR per $1000 0.90, 0.86–0.95, p = 0.01) and reimbursement (OR = 0.16, 0.03–0.76, p = 0.021), but not with the availability of national stroke strategies or reimbursement for other interventions. Similarly, increased delays until carotid endarterectomy were inversely associated with GDP per capita (OR per $1000 = 0.96, 0.93–0.99, p = 0.001), healthcare expenditure per capita (OR per $1000 = 0.76, 0.60–0.97, p = 0.03) and the number of centres per million population (OR = 0.73, 0.54–0.99, p = 0.047), but not with the presence of national stroke strategies or reimbursement for CEA (due to high levels of reimbursement).

## Discussion

Despite improvements in stroke care over the past decade,^3^ this estimation of current provision of secondary prevention identified significant reported gaps across Europe, with limited specialist follow-up, poor levels of adherence to medications and variable availability of advanced investigations such as prolonged cardiac monitoring. In lower income countries, respondents identified significant gaps in lifestyle management programmes, specialist assessment after TIA and monitoring for atrial fibrillation beyond ECG alone, whilst delays until assessment after TIA or treatment with carotid endarterectomy are often long.

Effective secondary prevention can reduce the risk of recurrent events by up to 80%.^3^ Tackling the gaps in the provision of well-established treatments identified in this survey could significantly improve care. For example, respondents estimated that post-stroke hypertension is rarely assessed by non-office-based monitoring and treatment is often deferred to primary care physicians, despite evidence that initiation of treatment in hospital increases medication use.^7^ These challenges may be met by novel strategies for BP control^12^ and by improved specialist follow-up for patients after cerebrovascular events, whilst increased reimbursement could increase availability of lifestyle management programmes. In lower income countries, increasing access to more prolonged cardiac monitoring than ECG alone may significantly reduce the burden of recurrent events due to AF,^13^ whilst expanding access to stroke specialists and vascular surgery should result in significant improvements in delays until assessment of TIAs and treatment of carotid stenosis. However, inequalities in secondary stroke prevention depended largely upon national income, representing a major societal challenge. Even here, identification of key gaps in care may enable targeting of limited resources to the most cost-effective interventions, including increasing availability of advanced interventions that are cost effective (DOACs^14^) or likely to be (prolonged cardiac monitoring,^13^ PFO closure^15,16^). Where feasible, gaps in interventions may be increased through healthcare policy, clinical guidelines and intervention-specific reimbursement, including strategic investment in training and development of capacity for more technical interventions.

Some assessments were reportedly performed in the majority of patients in some countries, but in few patients in others (transoesophageal echocardiography, intracranial vascular imaging, TIA clinics). This partly reflects differences in national trends (e.g. TIA clinics)^7^ or limited evidence for the clinical impact of some tests (TEE, TCD), warranting a need for further research before they can be recommended on a Europe-wide basis.

One strength of this study is the high number of respondents, reflecting the exceptional good-will of contributors and repeated requests for responses. Most respondents were leaders in national stroke societies with access to stroke registries and healthcare administrative data or provided expert national consensus opinion. However, there are limitations. Firstly, the authors did not have access to primary registry data and many responses were estimated. Therefore, the results are likely to be affected by opinions of respondents, resulting in unintentional biases. As such, significant differences between countries and identified determinants of quality of provision are indicative rather than definitive. Secondly, some respondents felt unable to provide a reliable response to some questions. Thirdly, we used 2016 IMF data to estimate GDP, which may have changed since 2016. Fourthly, multiple variables were combined into representative variables, potentially inflating inaccuracies in estimates. Finally, the number of centres performing specific procedures was standardised to the reported size of the population rather than the number of strokes occurring in each country. However, the resulting estimates were compared with the equivalent estimates standardised by the number of ischaemic strokes identified in the Global Burden of Disease report (unpublished).

This survey highlights gaps in secondary stroke prevention in more and less affluent European nations, identifying evidence-based, often cost-effective, development targets. These can be addressed through national and EU-wide policy initiatives, clinical guidelines, national and regional stroke strategies and provision of direct reimbursement for specific interventions. Although priorities in addressing these gaps will vary between countries, this survey provides the evidence to guide such priorities, focussing on interventions with a strong evidence base, including rapid assessment of TIA,^7^ limiting delays until carotid endarterectomy and maximising provision and maintenance of standard medical treatments. Furthermore, the survey demonstrated a lack of accurate healthcare data in many countries for secondary prevention. Establishing national and European-wide registries for monitoring the quality of stroke care is a key challenge of the next decade, without which appropriate policy development and targeting of research priorities is not feasible.

## Conclusions

Despite significant advances in secondary stroke prevention over the past decade, many gaps in the provision of routine, cost-effective, evidence-based interventions across Europe remain. Through identifying these gaps, the survey highlights the clinical, political and research priorities to improve provision of European secondary stroke prevention.

## Supplemental Material

Supplemental Material1 - Supplemental material for Availability of secondary prevention services after stroke in Europe: An ESO/SAFE survey of national scientific societies and stroke experts Click here for additional data file. Supplemental material, Supplemental Material1 for Availability of secondary prevention services after stroke in Europe: An ESO/SAFE survey of national scientific societies and stroke experts by A Webb, MR Heldner, D Aguiar de Sousa, EC Sandset, G Randall, Y Bejot, B van der Worp, V Caso, U Fischer and On behalf of the ESO-SAFE Secondary Prevention Survey Steering Group: on behalf of the Queen of Hearts and the RECONNECT consortia in European Stroke Journal

## Supplemental Material

Supplemental Material2 - Supplemental material for Availability of secondary prevention services after stroke in Europe: An ESO/SAFE survey of national scientific societies and stroke experts Click here for additional data file. Supplemental material, Supplemental Material2 for Availability of secondary prevention services after stroke in Europe: An ESO/SAFE survey of national scientific societies and stroke experts by A Webb, MR Heldner, D Aguiar de Sousa, EC Sandset, G Randall, Y Bejot, B van der Worp, V Caso, U Fischer and On behalf of the ESO-SAFE Secondary Prevention Survey Steering Group: on behalf of the Queen of Hearts and the RECONNECT consortia in European Stroke Journal

## Supplemental Material

Supplemental Material3 - Supplemental material for Availability of secondary prevention services after stroke in Europe: An ESO/SAFE survey of national scientific societies and stroke experts Click here for additional data file. Supplemental material, Supplemental Material3 for Availability of secondary prevention services after stroke in Europe: An ESO/SAFE survey of national scientific societies and stroke experts by A Webb, MR Heldner, D Aguiar de Sousa, EC Sandset, G Randall, Y Bejot, B van der Worp, V Caso, U Fischer and On behalf of the ESO-SAFE Secondary Prevention Survey Steering Group: on behalf of the Queen of Hearts and the RECONNECT consortia in European Stroke Journal
