# Supplementary material for: Availability of secondary prevention services after stroke in Europe: An ESO/SAFE survey of national scientific societies and stroke experts
Source: Eur Stroke J. 2018 Nov 27;4(2):110–8. doi: 10.1177/2396987318816136 (PMC6572590; doi:10.1177/2396987318816136)
Supplement: Supplemental Material1 - Supplemental material for Availability of secondary prevention services after stroke in Europe: An ESO/SAFE survey of national scientific societies and stroke experts [file Supplemental_Material3.pdf]

Country	Respondent 1 Name	Respondent 1 Position in National Stroke Society	Respondent 1 Affiliation	Respondent 2 Name	Respondent 2 Position in National Stroke Society	Respondent 2 Affiliation	Respondent 3 Name	Respondent 3 Position in National Stroke Society	Respondent 3 Affiliation
**Albania**	Petrela Mentor	Member	Mother Tereza University Hospital	Rroji Arben	Member	Mother Tereza University Hospital	Taka, Olsi	Member	Mother Tereza University Hospital
**Austria**	Stefan Kiechl	President	Medical University of Innsbruck	Franz Fazekas	Member	Medical University of Graz	Michael Brainin	Member	Danube University Krems
**Azerbaijan**	Rana Shiraliyeva	President of National Association of Neurologists	Azerbaijan State Advanced Training Institute for Doctors	Lala Tagizadeh		Clinical medical center (former City hospital N1)	Rahim Aliyev	Executive manager	Azerbaijan State Advanced Training Institute for Doctors
**Belgium**	André Peeters	President	Cliniques universitaires St. Luc	Geert Van Hooren	Secretary General	AZ St. Jan Brugge-Oostende	Jacques De Keyser	Past President	ZU Brussel VUB
**Bosnia / Herzegovina**	Ivan Perić		Health Department, Tomislavgrad						
**Bulgaria**	Ivan Milanov	Member of National Steering Committee	Medical University of Sofia	Dimitar Maslarov	Member of Steering committee	Medical University of Sofia, First MHAT	Staikov Ivan		Acibadem City Clinic Tokuda Hospital
**Croatia**	Hrvoje Budincevic	Croatian Stroke Society President	1) Sveti Duh University Hospital; 2) School of Medicine; University J.J. Strossmayer	Branko Malajcic	EC Member	1) University Hospital Center, 2) Zagreb School of Medicine,			
**Cyprus**	Costas Michaelides	n/a	American Medical Center	George Kaponides	n/a	Ygeia Polyclinic	Ktriakos Ktrallis	n/a	Appoloneid Hospital
**Czech Republic**	Ales Tomek	Chairman , Executive Committee	2nd Medical Faculty, Charles University	Robert Mikulik	Executive Committee	Masaryk University	Roman Herzig	Executive Committee	Medical faculty Hradec Králové, Charles University
**Denmark**	Helle K Iversen	Vice-chair	Righospitalet	Hanne Christensen	Chair	Bispebjerg Hospital	Søren Paaske Johnson	Member	Aalborg University and Aalborg University Hospital
**Estonia**	Janika Kõrv	Chair	Department of Neurology and Neurosurgery, Tartu University Hospital	Riina Vibo	Secretary	Department of Neurology and Neurosurgery, Tartu University Hospital	Siim Schneider	Member	Department of Neurology, North Estonia Medical Centre
**Finland**	Tiina Sarainen	Chair of The Finnish Stroke Society	Helsinki University Hospital	Daniel Strbian	NA	Helsinki University Hospital	Risto Roine	Finnish Brain Association, Chief Physician	Turku University Hospital

**France**	Serge Timsit	President SFNV	CHRU Brest, Université de Bretagne	Jean-Louis Mas	Past President SFNV	Hôpital Sainte-Anne, Université Paris-Descartes	Didier Leys	Former president of the SFNV (1998-2000)	Univ-Lille. Inserm
**Georgia**	Alexander Tsiskaridze	President	Department of Neurology, Pineo Medical Ecosystem, Tbilisi	Ioseb Burduladze	Director	Institute of Clinical Medicine	Sopio Sopromadze	Member	Pineo Medical Ecosystem
**Germany**	Martin Dichgans	President	Klinikum der Universität München, LMU, Munich	Joachim Röther	Past-President	Asklepios Klinik Altona	Matthias Endres	Past President	Charité-Universtitätsmedizin Berlin
**Greece**	Georgios Tsivgoulis	General Secretary of the Hellenic Society Of Cerebrovascular Diseases	Second Department of Neurology, “Attikon” Hospital University of Athens	Konstantinos Vadikolias	President	Department of Neurology, Democritus University of Thrace	Panayiotis Mitsias	Member	Department of Neurology Medical School, University of Crete
**Hungary**	Óváry Csaba	Board member	NICN	Daniel Bereczki	Past president	Semmelweis University, Department of Neurology	Szapáry László	President	Department of Neurology, University of Pecs
**Iceland**	Björn Thorarinsson		Landspitali University Hospital Reykjavik						
**Ireland**	Joseph Harbison	PI of National Register and National Audit	University of Dublin, Trinity College	Dominick McCabe	Committee Member, Irish National Clinical Advisory Group on Stroke Medicine,	Dept of Neurology, Adelaide and Meath Hospital; Trinity College Dublin, Ireland	Ronan Collins	National Clinical Lead in Stroke	University of Dublin, Trinity College
**Israel**	David Tanne	President, Israeli Neurology Association	Sheba Medical Center, Tel Aviv University	Natan Bornstein	Past President,	Shaare Zedek Medical Center	Ronen Leker	Member	Hadassah-Hebrew University Medical Center
**Italy**	Danilo Toni	President	University of Rome, Lasapienza	Simona Sacco	Member of the Board	University of L'Aquila	Maurizio Paciaroni	None	University of Perugia
**Kazakhstan**	Alma Zhusupova	President, Association of Neurology of Kazakhstan	Astana Medical University	Sholpan Nurmanova	Member	Astana Medical University	Zadash Muhambetova	Member	City outpatient department #6
**Kyrgyzstan**	Inna Lutsenko	Member	Kyrgyz State Medical Academy	Abdusalim Artykbaev	Chief	Freelance Neurol. Bishkek City Civil Clinic	Denis Titorenko	Member	Freelance Neurol. Bishkek City Civil Clinic

**Latvia**	Evija Miglane	President	P.Stradins Clinical University Hospital	Kristaps Jurjans	Member	P.Stradins Clinical University Hospital	Ilga Kikule	Board member	Riga East University Hospital
**Lithuania**	Aleksandras Vilionskis	Member of Council of Lithuanian stroke association	Republican Vilnius University Hospital,	Dalius Jatuzis	President of Lithuanian stroke association	Vilnius University, Faculty of Medicine	Daiva Rastenyte	Vice-president of Lithuanian stroke association	Lithuanian University of Health Sciences, the Hospital of LSMU “Kauno klinikos”
**Luxembourg**	Dirk Droste	Vascular working group of the neurology society	Centre Hospitalier de Luxembourg						
**Macedonia**	Anita Arsovska	Board member	University Clinic of Neurology	Maja Bozinovska-Smiceska	President	Special hospital 'St. Erasmo'	Elena Lickova	Member	Department of Neurology
**Moldova**	Stanislav Groppa	President of Moldavian Stroke Association	N.Testemiteanu State Medical/ Pharmaceutical University	Eremei Zota	Secretary of Moldavian Stroke Association	N.Testemiteanu State Medical /Pharmaceutica l University	Elena Manole		N.Testemiteanu State Medical /Pharmaceutical University
**Montenegro**	Mirjana Cukic		Neurology Clinic Clinical Centre of Montenegro	Ljiljana Radulovic		Neurology Clinic Clinical Centre of Montenegro	Zlatana Perovic		General Hospital Niksic
**Netherlands**	Diederik Dippel	Chair of national stroke work group	Erasmus MC, University medical center	Bart van der Worp	Member	UMC Utrecht	Yvo Roos	Member	AMC Amsterdam
**Norway**	Lars Thomassen	Leader	Haukeland University Hospital	Anne Hege Aamodt	Leader Norwegian Neurology Association	Oslo University Hospital	Martin Kurz	Treasurer	Stavanger University Hospital
**Poland**	Anna Czlonkowska	President of Cerebrovascular Section of Polish Neurological Society	Institute of Psychiatry and Neurology	Dariusz Gasecki	Co-worker of Polish Stroke Initiative	Medical University of Gdansk			
**Portugal**	Miguel Rodrigues	Treasurer (Portuguese Stroke Society)	Hospital Garcia de Orta	Elsa Azevedo	Vice-president (Portuguese Stroke Society)	Centro Hospitalar São João	Manuel Correia	President (Neurology Society)	Centro Hospitalar do Porto
**Romania**	Cristina Tiu	Future president (Romanian Society of Neurology	University hospital	Sorin Tuta	Vice-president	Institute of Cerebrovascula r Disorders	Ovidiu Bajenaru	Honorary president ad vitam	University hospital
**Russia**	Nikolay Shamalov		Pirogov Russian National Research Medical University,						

**Scotland**	Jesse Dawson	Lead, Stroke Research Network	University of Glasgow / NHSGGC	Fergus Doubal	Chair, Clinical Standards Committee	NHS Lothian			
**Serbia**	Ljiljana Beslać Bumbaširević	President of National Board for neurology	Neurology Clinic, Clinical Center of Serbia	Marija Žarkov	Member of National Board for neurology	Neurology Clinic, Clinical Center Vojvodina			
**Slovakia**	Peter Turcani	Member of executive committee	Department of Neurology, Comenius University, Bratislava						
**Slovenia**	Zvan Bojana	President of the National Stroke Society	University Medical Centre Ljubljana, Slovenia	Anita Resman Gaspersic		University Medical Centre Ljubljana, Slovenia			
**Spain**	María Alonso de Leciñana	Head of the Executive Committee	University Hospital La Paz	Óscar Ayo	Member of the Executive Committee	University Hospital Albacete	Mar Castellanos	Secretary of the Executive Committee	University Hospital A Coruña
**Sweden**	Michael Mazya	Chair, Swedish Acute Neurology Society	Karolinska University Hospital / Karolinska Institutet	Bo Norrving	Chair, Swedish Stroke Register	Skåne University Hospital / Lund University	Jesper Petersson		Skåne University Hospital / Lund University
**Switzerland**	Marcel Arnold	Chair	University Hospital, Bern	Leo Bonati	Coordinator Swiss Stroke Registry	University Hospital, Basel	Hakan Sarikaya	Co-chair of stroke task force	University hospital Bern
**Turkey**	Ethem Murat Arsava	General Secretary	Hacettepe University	Ibrahim Levent Gungor	Board Member	Ondokuz Mayis University	Kursad Kutluk	Former President	Dokuz Eylul University
**UK (exc Scotland)**	Fergus Doubal	Chair Clinical Standards Commitee	NHS Lothian	Thompson Robinson	President	University of Leicester			
**Ukraine**	Mykola Polishchuk	President	Shupik National Medical Academy of Post-Graduate Education	Tamara Mishchenko	Fellow	Karazin Kharkiv National University	Yuriy Flomin	Board member	Medical Center 'Universal Clinic "Oberig"'
**Uzbekistan**	Gafurov Bahtiyar	Chief, advanced training of doctors	Tashkent						
