# Supplementary material for: Availability of secondary prevention services after stroke in Europe: An ESO/SAFE survey of national scientific societies and stroke experts
Source: Eur Stroke J. 2018 Nov 27;4(2):110–8. doi: 10.1177/2396987318816136 (PMC6572590; doi:10.1177/2396987318816136)
Supplement: Supplemental Material2 - Supplemental material for Availability of secondary prevention services after stroke in Europe: An ESO/SAFE survey of national scientific societies and stroke experts [file Supplemental_Material4.pdf]

APPENIDX 2

ESO EUROPEAN STROKE ORGANISATION

SAFE

ESO/SAFE Survey on Secondary Prevention for Stroke

Name of the country/region you represent	Date of Completion

	First Respondent	Second Respondent	Third Respondent
**Name** *(Last, First):*			
**Career level:**	☐ Department Head<br>☐ Senior Physician	☐ Department Head<br>☐ Senior Physician	☐ Department Head<br>☐ Senior Physician
**Specialty**	☐ Neurology<br>☐ Stroke Physician<br>☐ Geratology<br>☐ Other ______________	☐ Neurology<br>☐ Stroke Physician<br>☐ Geratology<br>☐ Other ______________	☐ Neurology<br>☐ Stroke Physician<br>☐ Geratology<br>☐ Other ______________
**e-mail :**			
**European Stroke/Neurology organisation Member?**	☐ None ■ ESO<br>☐ Other ______________	☐ None ☐ ESO<br>☐ Other ______________	☐ None ☐ ESO<br>☐ Other ______________
**Position in national stroke organisation:**			
**Institution**			
**City**			

PART 1. STROKE REGISTRIES AND QUALITY CONTROL	
**In your country, do you have a stroke registry that includes secondary prevention measures?**	☐ National ☐ Hospital-based ☐ No ☐ Unknown<br>☐ For specific treatments only:<br>______________________________
**What proportion of stroke patients are covered by the registry?**	______________%
**If yes, which of the following characteristics apply to your registry/registries?** (Please tick all that apply)	☐ Informed consent ☐ Prospective<br>☐ Population-based ☐ Used for benchmarking<br>☐ Health administration data ☐ Available for research
**If yes, select which data can be derived from your registry**<br>(Please tick all that apply)	No of patients having:<br>☐ Ischaemic stroke ☐ TIA ☐ Haemorrhage<br>☐ MRI brain ☐ CT brain<br>☐ MRA ☐ Doppler ☐ CTA<br>☐ Endarterectomy ☐ Carotid stenting<br>☐ Cardiac monitoring ☐ PFO closure<br>No of patients prescribed:<br>☐ Antihypertensives ☐ Antiplatelets ☐ Statins<br>☐ Anticoagulation ☐ Novel oral anticoagulants<br>☐ Time to TIA assessment ☐ Follow up frequency
**If you use real-life data to complete this form (stroke registry / healthcare / extrapolation of local data), please describe this data source:**	

PART 2. ORGANISATION OF STROKE SYSTEMS OF CARE: SECONDARY PREVENTION	
**Which guidelines are used in your country for stroke?** *(Tick as many as apply)*	☐ ESO ☐ AHA/Stroke ☐ National ☐ Others ☐ None
**Do you have national guidelines covering secondary prevention after TIA or stroke?**	Yes ☐ No ☐ Unknown ☐
**In your country, at what level do any healthcare strategies operate to improve secondary stroke prevention?**	☐ No stroke specific strategic plans exist<br>☐ Country-wide ☐ Regional<br>☐ Hospital only ☐ For primary care<br>☐ Other______________
**Which specific secondary prevention** [illegible]	☐ Lifestyle management course ☐ Cardiac Monitoring (<72hr)<br>☐ Outpatient TIA Assessment ☐ Cardiac Monitoring (>72 hours)

PART 3. ASSESSMENT AFTER STROKE / TIA FOR SECONDARY PREVENTION

Question							
**What percentage of patients with TIA are formally assessed by:** *(if available, give number, otherwise select quintile by strikethrough)*	admission to hospital	____	☐0-20	■20-40	☐40-60	☐60-80	☐80-100
	the acute stroke service	____	☐0-20	☐20-40	☐40-60	☐60-80	☐80-100
**Is data from:** ☐ Estimation	a separate TIA clinic	____	☐0-20	☐20-40	■40-60	☐60-80	☐80-100
☐ Healthcare records ☐ Registry	a general medical clinic	____	☐0-20	☐20-40	☐40-60	☐60-80	☐80-100
	primary care only	____	☐0-20	☐20-40	☐40-60	☐60-80	☐80-100

Question				
**What is the guideline-recommended interval between referral and completion of assessment for TIA?**	☐ **None**	High-risk ______ days	Low-risk ______ days	***OR*** All *(if no distinction)* ______ days

Question							
**What percentage of patients with TIA are asessed within...**	Same day	______	☐0-20	☐20-40	☐40-60	☐60-80	☐80-100
**Is data from:** ☐ Estimation	Within 48 hours	______	☐0-20	☐20-40	☐40-60	☐60-80	☐80-100
☐ Healthcare records ☐ Registry	Within 1 week	______	☐0-20	☐20-40	☐40-60	☐60-80	☐80-100
	> 1 week	______	☐0-20	☐20-40	☐40-60	☐60-80	☐80-100

Question							
**What percentage of TIA/stroke patients undergo carotid imaging...**	by ultrasound	______	☐0-20	☐20-40	☐40-60	☐60-80	☐80-100
if available, give number, otherwise select quintile by strikethrough)	by CTA	______	☐0-20	☐20-40	☐40-60	☐60-80	☐80-100
**Is data from:** ☐ Estimation	by MRA	______	☐0-20	☐20-40	☐40-60	☐60-80	☐80-100
☐ Healthcare records ☐ Registry	by ≥2 modalities	______	☐0-20	☐20-40	☐40-60	☐60-80	☐80-100
	Not imaged	______	☐0-20	☐20-40	☐40-60	☐60-80	☐80-100

Question							
**What form of monitoring is most often used to exclude paroxysmal AF in appropriate patients?**	ECG only	______	☐0-20	☐20-40	☐40-60	☐60-80	☐80-100
	24-48 hr Holter	______	☐0-20	☐20-40	☐40-60	☐60-80	☐80-100
	>48 h monitor	______	☐0-20	☐20-40	☐40-60	☐60-80	☐80-100

Question							
**For secondary prevention purposes, what percentage of patients with ischaemic stroke / TIA undergo...**	Transthoracic Echo	______	☐0-20	☐20-40	☐40-60	☐60-80	☐80-100
	Transoesophageal Echo	______	☐0-20	☐20-40	☐40-60	☐60-80	☐80-100
**Is data from:** ☐ Estimation	Transcranial ultrasound	______	☐0-20	☐20-40	☐40-60	☐60-80	☐80-100
☐ Healthcare records ☐ Registry	Intracranial MRA / CTA	______	☐0-20	☐20-40	☐40-60	☐60-80	☐80-100
	Other______________________		☐0-20	☐20-40	☐40-60	☐60-80	☐80-100

Question						
**What percentage of patients have blood pressure monitoring after initial treatment after stroke by...**	Primary care only	☐0-20	☐20-40	☐40-60	☐60-80	☐80-100
	Hospital Clinic / BP	☐0-20	☐20-40	☐40-60	☐60-80	☐80-100
	Home / ambulatory device	☐0-20	☐20-40	☐40-60	☐60-80	☐80-100

Question		
**In your country, what is the usual target blood pressure after stroke?**	Age <70 ______ / ______mmHg	☐ All patients are treated regardless of BP
From: ☐ Guidelines ☐ Usual practice	Age >70 ______ / ______mmHg	

PART 4. MANAGEMENT

Question		
**Which lifestyle management programs are available to the majority of stroke patients as part of their medical care ?**	Combined risk factor management programs	☐
	Physical activity programs	☐
	Smoking cessation support	☐
	Weight loss programs	☐
	Other ____	

Question		
**If available, who usually provides these lifestyle management programs?**	Hospital stroke services	☐
	Primary care	☐
	Stroke-specific charitable organisations	☐

Question		Antiplatelet	Statin	Antihypertensive
**Where are these medical treatments usually initiated:**	Acutely by paramedic / GP	☐	☐	☐
	As inpatients / in TIA clinic	☐	☐	☐
	On discharge	☐	☐	☐
	In a follow-up clinic	☐	☐	☐
	Deferred to primary care	☐	☐	☐

Question							
**What percentage of patients following**	Antihypertensives	________	☐0-20	☐20-40	☐40-60	☐60-80	☐80-100

**What percentage of stroke patients at follow up (≥12 months) have:**<br><br>*if available, give exact number, otherwise select quintile by strikethrough)*<br>**Is data from:** ☐ Estimation<br>☐ Healthcare records ☐ Registry	BP recorded _____ ☐0-20 ☐20-40 ☐40-60 ☐60-80 ☐80-100<br>BP 'controlled' _____ ☐0-20 ☐20-40 ☐40-60 ☐60-80 ☐80-100<br>Cholesterol checked _____ ☐0-20 ☐20-40 ☐40-60 ☐60-80 ☐80-100<br>Statins _____ ☐0-20 ☐20-40 ☐40-60 ☐60-80 ☐80-100<br>Antiplatelets (non-AF) _____ ☐0-20 ☐20-40 ☐40-60 ☐60-80 ☐80-100<br>Anticoagulation (AF) _____ ☐0-20 ☐20-40 ☐40-60 ☐60-80 ☐80-100<br>DOACs (AF) _____ ☐0-20 ☐20-40 ☐40-60 ☐60-80 ☐80-100<br>Diabetic treatment _____ ☐0-20 ☐20-40 ☐40-60 ☐60-80 ☐80-100
**In your country, how many centres offer these interventions, and how many are performed yearly?**<br><br>**Is data from:** ☐ Estimation<br>☐ Healthcare records ☐ Registry	Centres / Performed<br>Carotid Endartectomy ______ __________<br>Carotid Stenting ______ __________<br>Vertebral / intracranial stents ______ __________<br>PFO closure ______ __________<br>Left atrial appendage closure ______ __________<br>Neurosurgery for AVMs ______ __________<br>Interventional treatment for AVMs ______ __________<br>Radiosurgery for AVMs ______ __________
**For patients needing carotid intervention, when does it happen?**<br>*if available, give number, otherwise select quintile by strikethrough)*<br>**Is data from:** ☐ Estimation<br>☐ Healthcare records ☐ Registry	Within 48 hours ______ ☐0-20 ☐20-40 ☐40-60 ☐60-80 ☐80-100<br>Within 1 week ______ ☐0-20 ☐20-40 ☐40-60 ☐60-80 ☐80-100<br>Within 2 weeks ______ ☐0-20 ☐20-40 ☐40-60 ☐60-80 ☐80-100<br>Within 1 month ______ ☐0-20 ☐20-40 ☐40-60 ☐60-80 ☐80-100<br>> 1 months ______ ☐0-20 ☐20-40 ☐40-60 ☐60-80 ☐80-100

**PART 5. FOLLOW UP FOR PREVENTION OF STROKE**	
**Which guidelines in use in your country recommend follow-up of patients with stroke?**	☐ ESO ☐ National ☐ None ☐ Other________________
**What proportion of patients are followed-up to for review of secondary prevention of stroke?**<br>*(if available, give exact number, or select quintile)*<br>**Is data from:** ☐ Estimation<br>☐ Healthcare records ☐ Registry	In hospital ____ ☐0-20 ☐20-40 ☐40-60 ☐60-80 ☐80-100<br>Dedicated clinic ____ ☐0-20 ☐20-40 ☐40-60 ☐60-80 ☐80-100<br>In primary care ____ ☐0-20 ☐20-40 ☐40-60 ☐60-80 ☐80-100<br>No follow up ____ ☐0-20 ☐20-40 ☐40-60 ☐60-80 ☐80-100
**If followed up in secondary care, how long are most patients with stroke followed up?**	No follow up ☐<br>≤6 weeks ☐<br>6-26 weeks ☐<br>6-12 months ☐<br>> 1 year ☐
**Who establishes stroke support groups in your country?**	Not commonly available ☐<br>Hospital ☐<br>Primary Care ☐<br>Stroke specific charities ________________ ☐
