# Supplementary material for: Availability of secondary prevention services after stroke in Europe: An ESO/SAFE survey of national scientific societies and stroke experts
Source: Eur Stroke J. 2018 Nov 27;4(2):110–8. doi: 10.1177/2396987318816136 (PMC6572590; doi:10.1177/2396987318816136)
Supplement: Supplemental Material3 - Supplemental material for Availability of secondary prevention services after stroke in Europe: An ESO/SAFE survey of national scientific societies and stroke experts [file Supplemental_Material5.pdf]

Availability of Secondary Prevention Services after Stroke in Europe: An ESO/SAFE survey of National Scientific Societies and Stroke Experts

Webb AJS[1], Heldner MR[2], Aguiar de Sousa D,[3] Sandset EC,[4] Randall G,[5] Bejot Y,[6] van der Worp HB,[7] Caso V,[8] Fischer U[2]

Webtable 1. Reported organisation of healthcare and research infrastructure in the secondary prevention of stroke. Groups are compared by chi-squared tests. N=total number of responses to the question; n=number of countries with availability of measures; GDP=gross domestic product; BP=blood pressure; TTE=transthoracic echocardiography; TEE= transesophageal echocardiography;TCD=transcranial ultrasound.

		N	GDP per capita<br>Lower tertile n (%)	Mid - Tertile n (%)	Upper tertile n (%)	All Respondents n (%)	p-value
Stroke Registry	National	45	1 (7)	4 (27)	9 (60)	14 (31)	0.006
	Hospital	45	6 (40)	8 (53)	8 (53)	22 (49)	0.7
	Population-based	34	3 (33)	3 (27)	6 (43)	12 (35)	0.71
	Health Admin	34	5 (50)	7 (70)	11 (79)	23 (68)	0.33
	Prospective	34	4 (40)	7 (70)	14 (100)	25 (74)	0.004
Guidelines	ESO	46	11 (69)	13 (87)	14 (93)	38 (83)	0.17
	AHA Stroke	46	10 (63)	11 (73)	13 (87)	34 (74)	0.31
	National	46	13 (81)	13 (87)	12 (80)	38 (83)	0.88
Healthcare Strategy	Country-wide	46	6 (38)	9 (60)	10 (67)	25 (54)	0.23
	Hospital only	46	6 (38)	2 (13)	4 (27)	12 (26)	0.31
	Regional	46	2 (13)	3 (20)	7 (47)	12 (26)	0.08
Reimbursement	Lifestyle Courses	44	4 (27)	0 (0)	7 (47)	11 (25)	0.015
	TIA clinics	45	9 (60)	12 (80)	14 (93)	35 (78)	0.09
	CEA	45	10 (67)	15 (100)	15 (100)	40 (89)	0.004
	Carotid Stenting	45	9 (60)	15 (100)	13 (87)	37 (82)	0.014
	LAA Closure	44	6 (40)	12 (86)	12 (80)	30 (68)	0.015
	Standard Drugs	45	14 (93)	15 (100)	15 (100)	44 (98)	0.36
	AF Monitor <72hr	45	9 (60)	15 (100)	14 (93)	38 (84)	0.005
	AF Monitor >72hr	45	6 (40)	6 (40)	10 (67)	22 (49)	0.24
	Loop recorder	45	3 (20)	7 (47)	10 (67)	20 (44)	0.036
	PFO Closure	45	7 (47)	15 (100)	13 (87)	35 (78)	0.001
Lifestyle Management	Combined	44	8 (53)	5 (36)	9 (60)	22 (50)	0.41
	Physical Activity	45	5 (33)	2 (13)	6 (40)	13 (29)	0.25
	Smoking	45	6 (40)	8 (53)	15 (100)	29 (64)	0.002
	Weight Loss	45	3 (20)	6 (40)	9 (60)	18 (40)	0.08

Webfigure 1. Reported reimbursement policies for investigation and treatment for the secondary prevention of stroke. Countries were grouped according to whether they reported that reimbursement was usually available for 'minimum medical treatments' (antiplatelets, statins, blood-pressure lowering) only, most 'standard investigations and interventions' (imaging, carotid endarterectomy) and whether the majority of advanced investigations and treatments were reimbursed (cardiac monitoring >72hrs, PFO closure, DOACs, novel antiplatelets, carotid stenting, left atrial appendage closure, implantable loop recorders).

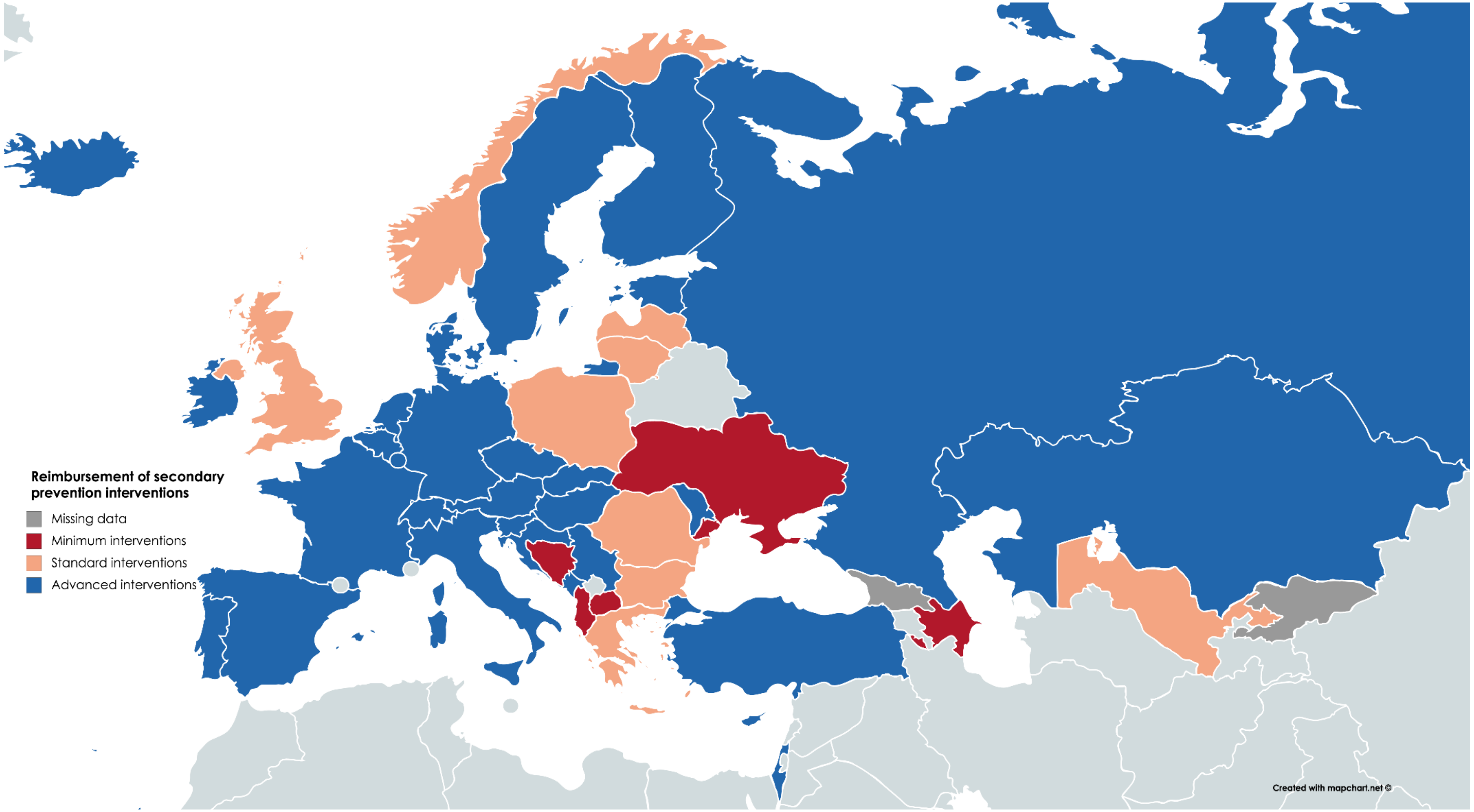

Webtable 2. Reported availability of services to >40% of patients for secondary prevention risk assessment after TIA or stroke, according to tertile of national income. Groups are compared by chi-squared tests. N=number of responses to the question; GDP=gross domestic product; BP=blood pressure; TTE=transthoracic echocardiography; TEE= transesophageal echocardiography;TCD=transcranial ultrasound.

				GDP per capita		All Respondents	
		N	Lower tertile n (%)	Mid - Tertile n (%)	Upper tertile n (%)	n (%)	p-value
TIA assessment location	Hospital	45	7 (44)	10 (67)	8 (57)	25 (56)	0.43
	Stroke Team	43	3 (20)	9 (64)	8 (57)	20 (47)	0.036
	TIA clinic	37	0 (0)	0 (0)	5 (36)	5 (14)	0.009
	General Clinic	41	4 (27)	0 (0)	1 (7)	5 (12)	0.09
	Primary Care	41	4 (27)	1 (8)	0 (0)	5 (12)	0.08
TIA assessment delay	Same Day	44	5 (31)	8 (53)	5 (38)	18 (41)	0.45
	Within 48hrs	44	6 (38)	6 (40)	6 (46)	18 (41)	0.89
	Within 1 week	44	3 (19)	0 (0)	5 (38)	8 (18)	0.031
	> 1 week	44	2 (13)	1 (7)	1 (8)	4 (9)	0.83
Carotid Imaging	Ultrasound	46	12 (75)	13 (87)	12 (80)	37 (80)	0.72
	CT-angiogram	46	4 (25)	5 (33)	8 (53)	17 (37)	0.25
	MR-angiogram	46	3 (19)	2 (13)	5 (33)	10 (22)	0.39
	2 modalities	43	3 (21)	3 (21)	5 (33)	11 (26)	0.70
Cardiac Monitoring	ECG only	42	12 (75)	5 (38)	2 (15)	19 (45)	0.005
	24-48 hours	45	9 (56)	13 (87)	11 (79)	33 (73)	0.14
	> 48 hours	42	0 (0)	2 (14)	4 (29)	6 (14)	0.10
BP monitoring	Primary care	41	8 (57)	13 (87)	12 (100)	33 (80)	0.017
	Hospital	42	4 (31)	6 (40)	4 (29)	14 (33)	0.79
	Out-of-office	39	9 (75)	7 (50)	4 (31)	20 (51)	0.09
Investigated with	TTE	45	9 (60)	12 (80)	8 (53)	29 (64)	0.28
	TEE	44	0 (0)	3 (20)	3 (21)	6 (14)	0.17
	TCD	44	2 (13)	5 (36)	1 (7)	8 (18)	0.11
	MRA / CTA	46	5 (31)	5 (33)	9 (60)	19 (41)	0.20

Webfigure 2. Reported delay until assessment of more than 60% of patients presenting with a TIA.

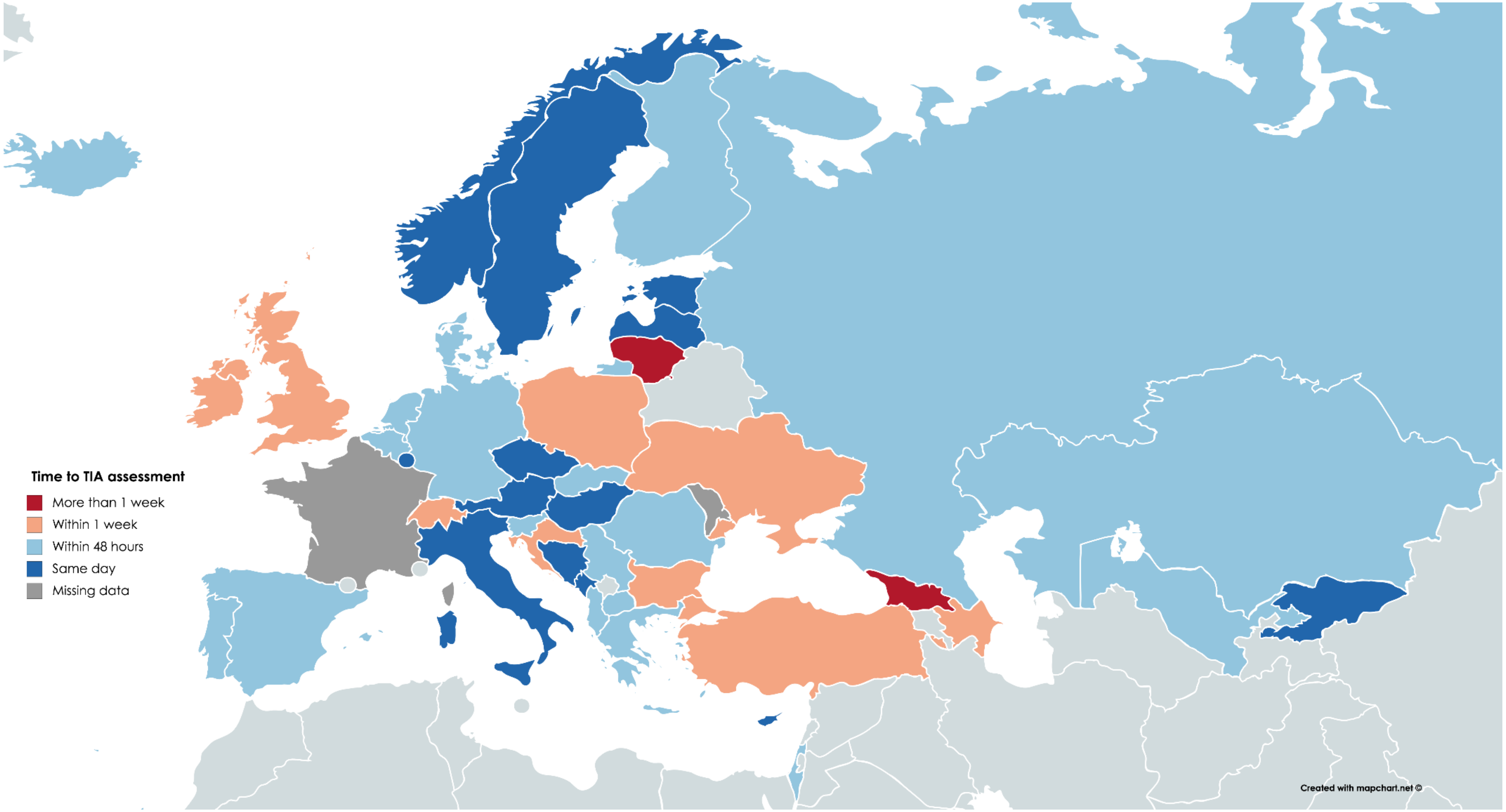

Webtable 3. Reported availability of services to >40% of patients for treatments for secondary prevention after TIA or stroke, according to tertile of national income. Groups are compared by chi-squared tests. N=number of responses to the question; GDP=gross domestic product; BP=blood pressure; TTE=transthoracic echocardiography; TEE= transesophageal echocardiography;TCD=transcranial ultrasound.

			GDP per capita			All Respondents	
		N	Lower tertile n (%)	Mid - Tertile n (%)	Upper tertile n (%)	n (%)	p-value
Initial treatment includes	BP-lowering	46	16 (100)	15 (100)	15 (100)	46 (100)	-
	Statin	46	12 (75)	14 (93)	15 (100)	41 (89)	0.07
	Antiplatelet	46	16 (100)	15 (100)	15 (100)	46 (100)	-
Carotid intervention	< 48 hours	36	0 (0)	1 (7)	1 (9)	2 (6)	0.61
	< 1 week	37	2 (18)	2 (14)	5 (42)	9 (24)	0.23
	< 2 weeks	39	3 (27)	8 (62)	10 (67)	21 (54)	0.11
	< 1 month	34	6 (55)	4 (31)	3 (30)	13 (38)	0.40
	> 1 month	33	6 (50)	0 (0)	0 (0)	6 (18)	0.002
Treatment at 1 year	BP measured	42	14 (88)	14 (100)	11 (92)	39 (93)	0.41
	BP controlled	43	12 (75)	13 (93)	11 (85)	36 (84)	0.42
	Lipids tested	42	12 (75)	14 (100)	10 (83)	36 (86)	0.14
	Statin	43	10 (63)	12 (86)	11 (85)	33 (77)	0.23
	Antiplatelet	43	15 (94)	14 (100)	12 (92)	41 (95)	0.59
	Anticoagulant	42	8 (50)	12 (86)	7 (58)	27 (64)	0.11
	DOAC	40	4 (27)	7 (50)	6 (55)	17 (43)	0.28
Follow up method	Hospital	43	6 (43)	5 (36)	8 (53)	19 (44)	0.63
	Specialist clinic	39	2 (17)	2 (17)	2 (13)	6 (15)	0.96
	Primary care	43	5 (36)	13 (93)	14 (93)	32 (74)	<0.001
	No follow-up	37	2 (17)	1 (9)	1 (7)	4 (11)	0.72
